# Effects of Persimmon (*Diospyros kaki* L. cv. Mopan) Polysaccharide and Their Carboxymethylated Derivatives on *Lactobacillus* Strains Proliferation and Gut Microbiota: A Comparative Study

**DOI:** 10.3390/ijms242115730

**Published:** 2023-10-29

**Authors:** Xiaowei Shen, Shanshan Xie, Huixin Zhang, Tao Wang, Bolin Zhang, Hongfei Zhao

**Affiliations:** Beijing Key Laboratory of Forest Food Processing and Safety, College of Biological Science & Biotechnology, Beijing Forestry University, Beijing 100083, China; shenxiaoweigirl@163.com (X.S.); xiess0907@126.com (S.X.); zhanghx923@163.com (H.Z.); wtshro@163.com (T.W.); zhangbolin888@163.com (B.Z.)

**Keywords:** carboxymethyl modification, persimmon, polysaccharides, *Lactobacillus*, gut microbiota

## Abstract

Persimmon is a fruit that contains sugars, vitamins, phenolic compounds, and various other nutrients. The aim of this study was to explore the structure of carboxymethylated persimmon polysaccharide (CM-PFP) and its interaction with the human gut microbiota. Carboxymethyl modification of the persimmon polysaccharide (PFP) increased both the Mw and Mn, enhanced dispersion stability, and decreased thermal stability. Both PFP and CM-PFP promoted the proliferation of *Lactobacillus* while inhibiting the proliferation of *Staphylococcus aureus* and *Escherichia coli*. In the simulated fecal fermentation, the pH of PFP- and CM-PFP-containing media decreased, the content of short-chain fatty acids increased, and the abundance of intestinal flora at the phylum and genus levels changed. The relative abundance of harmful intestinal bacteria was significantly reduced in both PFP and CM-PFP groups. Furthermore, it was found that CM-PFP was more easily metabolized than PFP, glucose, and fructo-oligosaccharide (FOS) and had a proliferation increase effect on *Lactobacillus*. Therefore, CM-PFP has a significant positive effect on both *Lactobacillus* proliferation and the human gut microbiota.

## 1. Introduction

Persimmon (*Diospyros kaki L.* cv. Mopan) is a widely cultivated fruit globally, especially in China, America, Japan, and Korea. Mopan persimmon is the main species in northern China and is mainly produced in Fangshan, Beijing, and Mancheng, Hebei Province. The Mopan persimmon is a fruit that contains high levels of sugars, vitamins, phenolic compounds, and various other nutrients [1]. Presently, research conducted on persimmons has centered on polysaccharides, polyphenols, tannins, proanthocyanidins, and their derivatives [2,3,4]. Polysaccharides are active substances with low toxicity and good biocompatibility. Research on polysaccharides found in persimmons has primarily centered on their potential for modification and their abilities as antioxidants, anti-inflammatory agents, antiwrinkle remedies, and anticoagulants [5,6,7]. Previous studies reported that persimmon polysaccharides significantly positively affect *Lactobacillus* proliferation and maintain the balance of intestinal microorganisms in mice [8]. Similarly, longan pulp polysaccharides, Jujube (*Ziziphus jujuba* Mill.) polysaccharides, and Maitake polysaccharides have shown similar positive effects on *Lactobacillus* proliferation [9,10,11].

Polysaccharides have biological activity that stems from their chemical structure, and as such, the development of novel polysaccharide derivatives via chemical modification is a hot topic. Chemical modifications such as sulfation, acetylation, carboxymethylation, and phosphorylation are frequently employed to modify the structure of polysaccharides. For example, sulfation was found to be effective in increasing the anticoagulant activity of persimmon polysaccharides [6]. As reported by Wang et al., the parent polysaccharide β-glucan from *Poria cocos* sclerotium was devoid of anticancer activity, but its sulfated, carboxymethylated, methylated, hydroxyethylated, and hydroxypropylated derivatives exhibited anticancer activity in S180 cells and gastric cancer cells [12]. Carboxymethylation introduces carboxymethyl groups onto the polysaccharide chain, which increases the solubility of the polysaccharide and potentially alters its biological activity [13]. After carboxymethylation, bitter gourd polysaccharide could possess DPPH radical- and superoxide anion-scavenging ability and can inhibit lipid peroxidation [14]. Carboxymethylated degraded *Sargassum fusiforme* polysaccharides show significant antibacterial activity against *Staphylococcus aureus* (*S. aureus*), *Bacillus subtilis*, *Escherichia coli* (*E. coli*), *Salmonella* spp. and *Pseudomonas Aeruginosa* [15]. Carboxymethylation on polysaccharides isolated from the medicinal plant *Cyclocarya paliurus* possesses antitumor activities and protective effects on cell oxidative stress [16]. However, not every chemical modification significantly improves the bioactivity of polysaccharides, which are related to a large number of structural features, including the functional groups, degree of polymerization or branching, glycosidic bonds, and stereochemical conformation of the polysaccharide [17].

The structural identification and properties pertaining to the physical and chemical characteristics of PFP and CM-PFP remain currently ambiguous. Additionally, how the carboxymethylation of persimmon polysaccharides affects the human intestinal flora is not yet fully understood and requires further investigation. Therefore, the objectives of this study were to: (1) determine the physicochemical properties and structural characterization of PFP and CM-PFP; (2) explore the prebiotic activity of PFP and CM-PFP on the growth of *Lactobacillus*; (3) analyze how PFP and CM-PFP impact physiology and composition of gut microbiota in vitro.

## 2. Results

### 2.1. Chemical Composition of PFP

**Figure 1:**
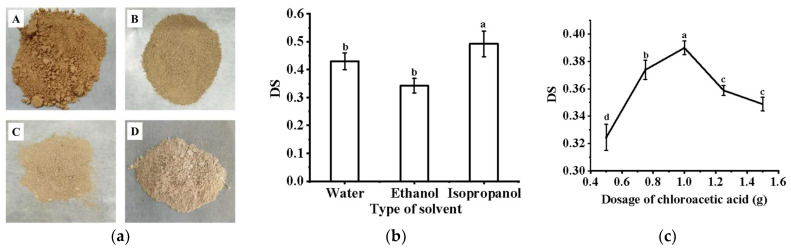

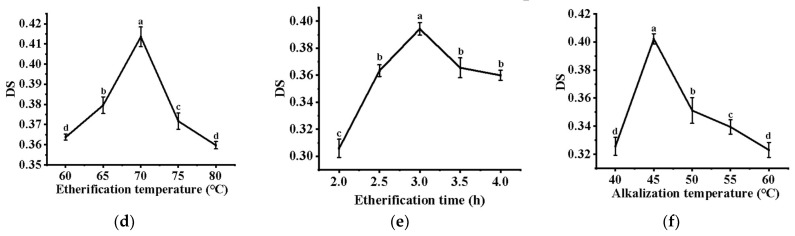
(**a**) The appearance characterization of Crude PFP (A), Deproteinizated PFP (B), PFP (C), CM-PFP (D); (**b**) Effect of solvent type on carboxymethylation effect; (**c**) Effect of chloroacetic acid dosage on carboxymethylation effect; (**d**) Effect of etherification temperature on carboxymethylation effect; (**e**) Effect of etherification time on carboxymethylation effect; (**f**) Effect of alkalization temperature on carboxymethylation (Different lowercase letters indicate statistical significance was determined using analysis of variance (ANOVA), and differences were considered statistically significant when *p* < 0.05.).

The extraction yield of crude PFP was 13.8 ± 0.03%, not much different from the results of previous research (12.51%) [8]. The chemical compositions of the PFP under different treatments are shown in Table 1. The total sugar content of crude PFP was 42.8 ± 0.21%, and the sugar content of deproteinized PFP was 64.3 ± 0.33%. The sugar content of PFP was 77.24 ± 0.07%, and the protein content was 2.9 ± 0.41%. After carboxymethyl modification, there were no significant differences in the sugar and protein contents of the modified persimmon polysaccharides. The different extraction and modification methods affected the color of the polysaccharides (Figure 1a). As the refining process of persimmon polysaccharides was optimized, the overall color transitioned to white, the red color weakened, and the degree of yellow slightly increased (Table 1).

### 2.2. Analysis of Carboxymethylation Modification of PFP

Using different solvents affected the degree of substitution (DS) of CM-PFP. As shown in Figure 1b, using isopropanol as the reaction solvent yielded the best result, with a DS of 0.493 (*p* < 0.05). The DS of CM-PFP was further increased using a higher dose of chloroacetic acid (Figure 1c), and the maximum DS was 0.390 at 1 g (*p* < 0.05). However, at high chloroacetic acid dosages, the DS of CM-PFP slightly decreased. A similar effect was observed when varying the etherification temperature. At first, as the temperature increased, the DS of PFP also increased; however, at higher temperatures, the DS started to decrease (Figure 1d). When the etherification temperature was 70 °C, the DS of PFP reached the maximum of 0.414 (*p* < 0.05). Additionally, the DS values of CM-PFP initially exhibited an increase as etherification time increased (Figure 1e), and when the etherification time was 3.5 h, the DS of CM-PFP reached its highest value (*p* < 0.05). However, an etherification time of longer than 3.5 h reduced the carboxymethylation. Furthermore, as the alkalization temperature was initially increased, the level of DS increased and reached a maximum value of 0.402 (*p* < 0.05) at an alkalization temperature of 45 °C (Figure 1f). As shown in Table S1a, the specific order of the influential factors was A (alkalization temperature) > C (etherification temperature) > D (etherification time) > B (chloroacetic acid dosage). From these experiments, the optimal conditions were selected: alkalization temperature of 40 °C; chloroacetic acid dosage of 1 g; etherification temperature of 55 °C and etherification time of 2.5 h. Implementation of the optimized conditions to carboxymethylation in isopropanol significantly affected the DS (Table S1b).

### 2.3. Physicochemical Properties and Structural Characterization of PFP and CM-PFP

#### 2.3.1. The Fourier Transform Infrared (FTIR) Spectra of PFP and CM-PFP Analysis

**Figure 2:**
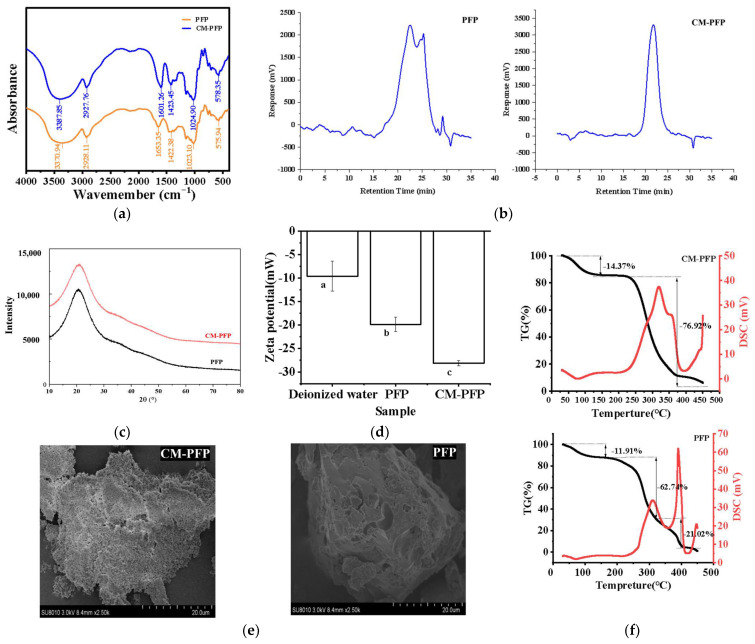
(**a**) FTIR spectra of PFP and CM-PFP; (**b**) GPC chromatogram of PFP and CM-PFP for molecular weight determination; (**c**) X-ray diffractogram spectrum of PFP and CM-PFP; (**d**) Zeta-potentials of PFP and CM-PFP solutions; (**e**) SEM pictures of PFP and CM-PFP; (**f**) Thermal stability image of PFP and CM-PFP (Different lowercase letters indicate statistical significance was determined using ANOVA, and differences were considered statistically significant when *p* < 0.05.).

The FTIR spectra of PFP and CM-PFP are shown in Figure 2a. The PFP spectrum contained absorption bands at 3370 cm^−1^, 2928 cm^−1^, and 1023 cm^−1^, which are characteristic of persimmon polysaccharides. The peak at 3370 cm^−1^ was attributed to the -OH vibration absorption peak, a typical polysaccharide peak. The stretching vibration peak of the C-H bond of the saccharide was near 2928 cm^−1^, and the peak at 1023 cm^−1^ revealed the asymmetric vibrations of the C-O-C and C-O-H glycosidic ring. The spectrum was consistent with a previous FTIR spectrum of PFP that contained strong OH stretching at 3200–3500 cm^−1^ and strong OH bending at 1620–1660 cm^−1^ [5]. In the FTIR spectrum of CM-PFP, new vibration maxima at 1423 cm^−1^ and 1601 cm^−1^ indicated that carboxymethyl groups existed. The band near 1423 cm^−1^ was attributed to the symmetrical stretching vibration of the COO- of the carboxymethyl group. The asymmetric stretching vibration of the C=O bond of the carboxymethyl group was responsible for the peak at 1601 cm^−1^.

#### 2.3.2. The Mw of PFP and CM-PFP Analysis

For the structural characterization of natural polysaccharides, Weight-average Molecular Weight (Mw) is regarded as an important element. Number-average Molecular Weight (Mn) refers to the result of the statistical average of the molecular number distribution. The polydispersity index, PD (Mw/Mn), indicates the degree of molecular weight dispersion. The Mw distributions of PFP and CM-PFP are shown in Figure 2b. A single symmetrical peak indicated that CM-PFP was a homogeneous mixture. Furthermore, the results indicate that the majority of PFP compounds have an Mw in the 0–5000 Da range, which accounted for 51.668% of the molecules. Understandably, an increase in mass was observed after carboxymethylation, and the majority of CM-PFP compounds were found within a mass range of 5000–10,000 Da, which accounted for 64.506% of the compounds (Table 2). This was further illustrated in the PD values of PFP and CM-PFP, which were 6.126 and 1.743, respectively. A larger PD value indicates a more scattered distribution of Mw.

#### 2.3.3. Monosaccharide Composition of PFP and CM-PFP Analysis

Our results show that the PFP and CM-PFP monosaccharide compositions were essentially the same and that the proportion of each monosaccharide remained unchanged (Table 3). Hence, the backbone structure of the polysaccharides remained intact when functional groups were introduced. PFP and CM-PFP contained mainly Ara, Gal, Glc, Man, and Rib. Notably, Glc may form the backbone of the main chains of PFP and CM-PFP as the Glc content of PFP was 95.62%, and the content of CM-PFP was 97.61%.

#### 2.3.4. X-ray Diffraction (XRD)

XRD is used to determine the crystallographic structure of polysaccharides and their physical properties, such as flexibility, dissolubility, and swelling. As shown in Figure 2c, the results show that a broad and low-intensity diffraction peak appeared at the angle of 2θ = 20°.

#### 2.3.5. Zeta Potential of PFP and CM-PFP

The zeta potential is an essential parameter for the dispersion characteristics of colloidal systems. It describes the nature of the charge on the surface of colloidal particles and can be used to analyze the interactions of charged particles in solution [18]. Higher zeta potential values represent stable solutions owing to the repulsion between highly charged particles, whereas lower zeta potential values reflect intermolecular forces overcoming repulsive forces to cause macromolecule aggregation [19]. As shown in Figure 2d, the Zeta potentials of PFP and CM-PFP were −19.88 ± 1.49 mV and −28.18 ± 0.54 mV, respectively. In this study, the absolute value of the zeta potential of PFP was low; therefore, the intermolecular repulsion was less than the attraction, and the molecules aggregated easily.

#### 2.3.6. Thermal Stability Analysis of PFP and CM-PFP

Through Thermogravimetric (TG) analysis and differential scanning calorimetry (DSC) analysis, it was observed that the residual water in the polysaccharide was evaporated at 50–150 °C, with a mass loss of 11.91% for PFP and 14.37% for CM-PFP (Figure 2f). Then, a more rapid weight loss of 62.74% occurred between 50 °C and 320 °C, as a result of the structural depolymerization of PFP. As the temperature increased to the third temperature range, a mass loss of 21.02% was observed for PFP. Conversely, significant degradation of polysaccharide molecules occurred between 150–380 °C for CM-PMP, with a mass loss of 76.92%. PFP exhibited a small exothermic peak at 80 °C and two endothermic peaks at 310–380 °C, while CM-PFP displayed an exothermic peak at 100 °C and an endothermic peak at 325 °C. The weight loss of CM-PFP when the temperature increased to 150 °C was higher than the weight loss of PFP over the same range. Combined with the TG-DSC analysis, the mass loss was greater during the structural degradation stage, indicating a decrease in the thermal stability of CM-PFP compared to PFP.

#### 2.3.7. Scanning Electron Microscopy (SEM) of PFP and CM-PFP

SEM showed that PFP had a rough surface with varying pore sizes and highly branched, interleaved structures with a number of sheet-like or chain-like shapes (Figure 2e). The surface of CM-PFP was composed of numerous sheets stacked on top of one another and packed granular aggregates that were loose and porous. Compared to the micrograph of PFP, the surface of CM-PFP was more porous.

### 2.4. The Effect of PFP and CM-PFP on Lactobacillus Proliferation

**Figure 3:**
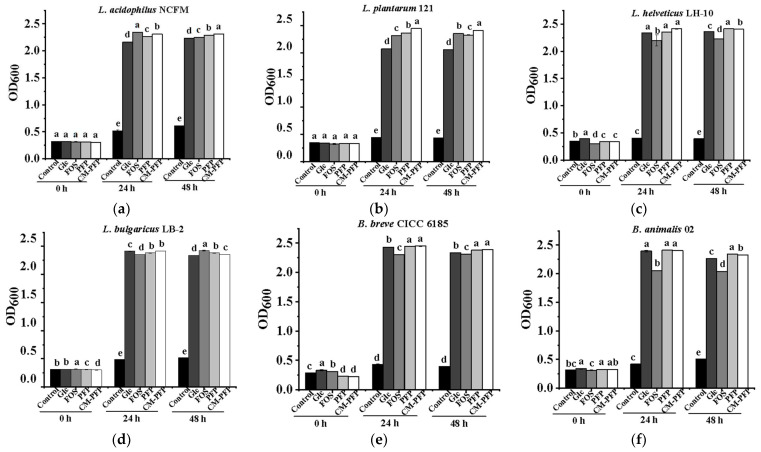
(**a**–**f**) Effects of MRS media from different carbon sources on the proliferation of selected *lactobacillus* (Different lowercase letters indicate significant differences among MRS media according to ANOVA test (*p* < 0.05)).

Compared to the control group, Glc, PFP, CM-PFP, and FOS significantly promoted the growth of the six *Lactobacillus* bacteria assessed after 48 h of culture (*p* < 0.05) (Figure 3a–f). The compounds were ordered as follows in terms of the effect of promoting growth: CM-PFP > PFP > Glc > Control. For *L. acidophilus* NCFM and *L. plantarum* 121, PFP and CM-PFP were significantly more proliferative than Glc and controls. However, after 24 h of culture, the FOS group had a more proliferative effect than PFP but less effective than CM-PFP (*p* < 0.05). In addition, *L. helveticus* LH-10 and *L. bulgaricus* LB-2 proliferated significantly better in PFP and CM-PFP media compared to FOS media (*p* < 0.05). The same results were observed for *B. animals* 02 and *B. breve* CICC 6185. Additionally, the proliferation of *L. bulgaricus* LB-2 was significantly lower on PFP and CM-PFP media than on Glc media (*p* < 0.05). After 48 h, the growth of the six *Lactobacillus* strains in PFP and CM-PFP media was considerably superior to that in the Glc group (*p* < 0.05). With the exception of *L. bulgaricus* LB-2, *Lactobacillus* strains grown on CM-PFP media were significantly more than those grown on FOS media (*p* < 0.05).

The prebiotic activities of the different carbon sources were evaluated by comparing the proliferation index (PI) of *Lactobacillus* cultured for 48 h. Our results show that the PI of the six *Lactobacillus* species remained stable after 24 h of culture (Table S2a). The proliferation of the six *Lactobacillus* species after 48 h in PFP and CM-PFP media was higher than in Glc media (*p* < 0.05), and similar to the proliferation in FOS media. Furthermore, CM-PFP was markedly more effective at *Lactobacillus* proliferation than PFP (*p* < 0.05). After 48 h of incubation, PI decreased slightly. The overall effect of PFP and CM-PFP on proliferation after 48 h was substantially greater than the Glc media. (*p* < 0.05). Meanwhile, except for *L. helveticus* LH-10, the other five *Lactobacillus* strains proliferated substantially more on CM-PFP media than on PFP media (*p* < 0.05).

The pH values of the six *Lactobacillus* strains decreased significantly after 48 h of culture (Table S2b). Except for *L. plantarum* 121, the PFP and CM-PFP media had lower pH than the Glc media (*p* < 0.05). Meanwhile, the pH of *L. plantarum* 121 and *L. helveticus* LH-10 remained at a lower level in the CM-PFP medium compared with the PFP medium (*p* < 0.05). The decrease in pH was mainly due to the acid produced during anaerobic fermentation.

### 2.5. Inhibitory Effects on Escherichia coli and Staphylococcus aureus

**Figure 4:**
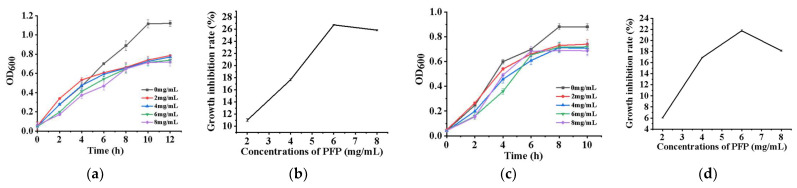
(**a**) The growth curve of *E. coli* with different concentrations of PFP; (**b**) Growth inhibition rate of *E. coli* with different concentrations of PFP; (**c**) The growth curve of *S. aureus* with different concentrations of PFP; (**d**) Growth inhibition rate of *S. aureus* with different concentrations of PFP.

The inhibition experiments revealed that the OD_600_ of *E. coli* and *S. aureus* was less than that of the control group in the addition of PFP following 6 h of culture (Figure 4a,c). The inhibition of *S. aureus* and *E. coli* reached the maximum at 6.00 mg/mL of PFP in the growth media; the inhibition maxima were 26.71% and 21.75%, respectively (Figure 4b,d). These results indicated that PFP displayed inhibitory activity and sensitivity effects on *E. coli* and *S. aureus.* Antibacterial activity decreased as the PFP concentration was exceeded. The excessive PFP might be hydrolyzed by the *Lactobacillus* and is available for use.

Inhibition of *E. coli* and *S. aureus* growth by different carbon sources is shown in Table S2c. At 6.00 mg/mL polysaccharide concentration, PFP displayed the best inhibitory effect on *E. coli* (28.83 ± 0.193%), and CM-PFP displayed the best inhibitory effect on *S. aureus* (18.536 ± 0.038%). Both PFP and CM-PFP had greater inhibitory effects on *E. coli* than on *S. aureus*. FOS had the worst inhibitory effect on both of the bacteria tested and promoted *S. aureus* to grow. The results indicated that various carbon sources inhibit *E. coli*. and *S. aureus* differently at the same concentration, likely linked to the polysaccharide structure.

### 2.6. In Vitro Fecal Fermentation

#### 2.6.1. SCFAs Produced in PFP and CM-PFP Medium

**Figure 5:**
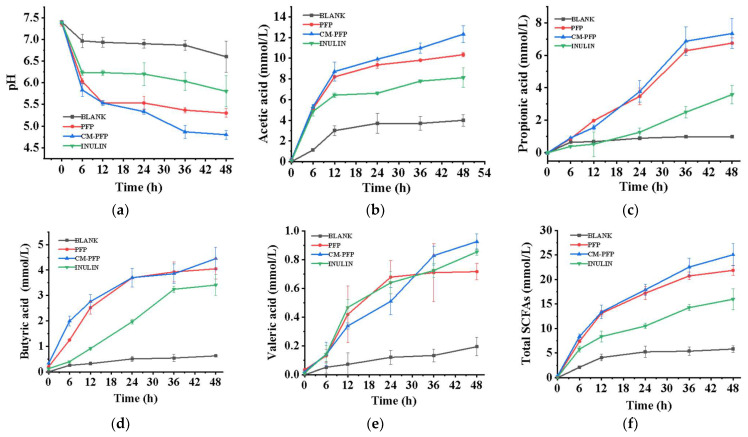
Changes in pH (**a**), acetic acid (**b**), propionic acid (**c**), butyric acid (**d**), valeric acid (**e**), total SCFAs (**f**) of the fermentation liquor during in vitro fermentation.

During the fermentation, the pH decreased significantly between 0 and 12 h before stabilizing between 12 and 48 h (Figure 5a). This result is consistent with that of a study of fecal flora grown on rice with FOS [20]. Compared to the negative control, the pH of the experimental groups was considerably reduced (*p* < 0.05). The pH of the fermentation with CM-PFP was the lowest and significantly lower than that of PFP. The levels of acetic acid, propionic acid, butyric acid, and valeric acid increased markedly in the CM-PFP treatment after 48 h of in vitro fermentation (Figure 5b–e). The total SCFA concentration of each type of polysaccharide treatment showed a substantial increase over the control (*p* < 0.05) (Figure 5f).

#### 2.6.2. Phylum-Level Species Relative Abundances

**Figure 6:**
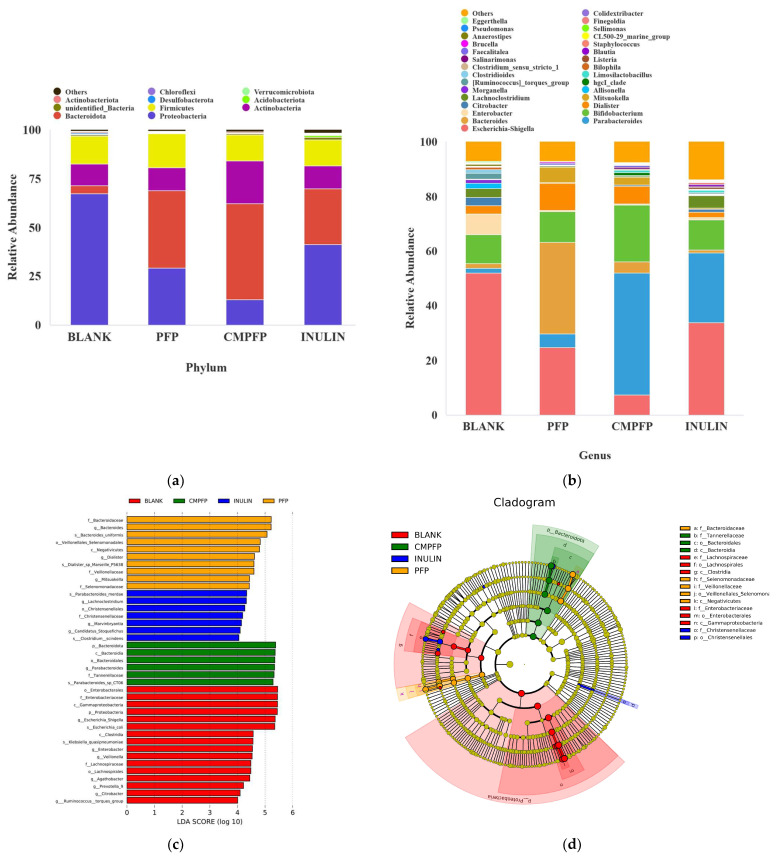
(**a**) Relative abundance of microbes in phylum; (**b**) Relative abundance of microbes in genus; (**c**) Histogram of the LDA scores across groups after 48 h of in vitro fecal fermentation; (**d**) Taxonomic cladogram across groups after 48 h of in vitro fecal fermentation.

At the phylum level, the main microorganisms in the control group were *Proteobacteria* (67.38%), *Firmicutes* (15.08%), *Bacteroidetes* (4.13%), and *Actinobacteria* (10.89%) (Figure 6a). Compared to the control group, a significantly higher relative abundance of *Bacteroidetes* was observed in the PFP, CM-PFP, and Inulin groups, with the order of increase as follows: CM-PFP (49.03%) > PFP (39.79%) > Inulin (28.54%). In addition, there was an improvement in the relative abundance of *Firmicutes* (17.49%) in the PFP group in comparison with the control. PFP and CM-PFP increased the *Bacteroidetes*/*Firmicutes* ratio from 0.28 to 2.28 and 3.67, respectively, higher than the Inulin group (2.1). Furthermore, the abundance of *Actinobacteria* was raised in the CM-PFP group (22.08%) over the control group. However, the relative abundance of *Actinobacteria* (11.65%) and *Firmicutes* (13.59%) in the Inulin group was similar to control. The relative abundance of *Proteobacteria* (41.29%) reduced while that of *Bacteroidetes* (28.54%) improved in the Inulin group. It also was found that the Inulin group was richer in *Proteobacteria* [21].

#### 2.6.3. Genus-Level Species Relative Abundance

We found that the control group was mainly composed of *Escherichia* (52.01%), *Parabacteroides* (1.68%), *Bacteroides* (1.72%), *Bifidobacterium* (10.56%), *Enterobacter* (7.52%), *Dialister* (3.01%), *Citrobacter* (3.13%), and *Clostridium* (3.24%) (Figure 6b). In the Inulin group, *Parabacteroides* (25.5%) increased, and the harmful intestinal bacteria *Escherichia*, *Enterobacter*, and *Citrobacter* decreased. Additionally, an increase in the relative abundance of *Dialister* was found in the Inulin group. The relative abundance of *Bacteroides* in the PFP group significantly increased (33.64%). Meanwhile, the levels of *Parabacteroides* (44.49%) and *Bifidobacterium* (20.79%) in the CM-PFP group were higher than those in the control group. In addition, the relative abundance of harmful intestinal bacteria such as *Escherichia*, *Enterobacter*, *Citrobacter*, and *Clostridium* was significantly lower in the PFP and CM-PFP groups than in the control group.

#### 2.6.4. Linear Discriminant Analysis Effect Size (LEfSe) Abundance Difference Analysis

Through LEfSe analysis, it was found that a total of 37 genera had linear discriminant analysis (LDA) scores above 4.0, with significant differences between groups (Figure 6c). There were 16, 7, 6, and 10 dominant genera in the control, PFP, CM-PFP, and Inulin groups, respectively. As shown in Figure 6d, 6, 5, 3, and 2, dominant species were detected in the control, PFP, CM-PFP, and Inulin groups, respectively. *Veillonellaceae* was the dominant bacteria in the PFP group, and *Bacteroides* and *Parabacteroides* were the dominant bacteria in the CM-PFP group. *Enterobacteriaceae* population was substantially suppressed in comparison with the control group, including *Salmonella*, *E. coli*, *Shigella*, and other pathogenic bacteria.

## 3. Discussion

### 3.1. Carboxymethylation of PFP

The PFP and CM-PFP can be better dissolved in isopropanol so that the reaction is uniform and stable. Simultaneously, isopropanol effectively transferred the heat and mass so that fewer side reactions occurred and the utilization rate of the etherification reagent was higher. The chloroacetic acid dosage affected the pH of the entire reaction system. More chloroacetic acid results in more CH_2_COO^−^, which increases the probability of polysaccharide reaction. However, if the chloroacetic acid dosage is too high, the pH value of the entire reaction system would be reduced, affecting the overall reaction [22]. The reaction between chloroacetic acid and the sodium salt of the polysaccharide proceeded more efficiently as the etherification temperature increased. In addition, the interaction between carboxymethyl and hydroxyl groups increased, thus promoting the formation of carboxymethylated polysaccharides [23]. An increase in alkalization temperature may result in the degradation of the polysaccharides and lead to carboxymethylation modification under strong alkali conditions. Subsequently, the color of the product became darker because of the reduction in DS and increased side reactions. Increasing the alkalization time can create a better environment for the carboxymethylation reaction and increase the probability of a reaction occurring. However, when the alkalization time was too long, the polysaccharide was destroyed by the strong alkali conditions, and the long chain of the polysaccharide was broken, resulting in a decreasing trend in the overall substitution degree [24].

### 3.2. Physicochemical Properties and Structural Characterization of PFP and CM-PFP

Additionally, the FTIR spectra of PFP and CM-PFP were similar, indicating that carboxymethylation did not change the PFP backbone structure. The region between 1200 cm^−1^ and 900 cm^−1^ contained bands belonging to the fingerprint region of carbohydrates and was dominated by ring vibrations overlapped with the stretching vibration of ν(C-OH) side groups and the ν(C-O-C) glycosidic bond [25]. There were three absorption peaks in the 1200–1000 cm^−1^ range, indicating that PFP was a pyran-type sugar ring [22]. Another promising finding was that the bands of CM-PFP in the fingerprint region remained unaltered, indicating that the PFP backbone remained intact. Therefore, FTIR analysis demonstrated the successful carboxymethylation modification of PFP.

The change in Mw was largely due to the breakdown and loss of low-molecular-weight carbohydrate chains during the modification process. High-molecular-weight fractions of persimmon extract contain large amounts of the uronic acid Gal A and low levels of neutral sugars, such as Ara, Gal, Glc, Xyl, Rha, and Man [7]. The neutral heteropolysaccharides in persimmon peel are made up of Fuc, Rha, Ara, Gal, Glc, Xyl, and Man [26]. Unlike the above studies, Xyl and Rha were not detected in the PFP in the present study. Yang et al. also did not report Xyl or Rha, but the contents of Man, Glc, Gal, and Ara were different from the present work [8]. Furthermore, chemical modifications may cause hydrolysis of natural polysaccharides, increasing the uronic acid content [16]. However, a lower content of uronic acid was found in CM-PFP compared with PFP. Differences in polysaccharide extraction and modification methods may account for the differences in monosaccharide composition.

There were no other diffraction peaks that indicate that PFP and CM-PFP have an amorphous structure and that carboxymethyl modification does not change the crystal structure of PFP. This is similar to the crystal forms of most plant polysaccharides, such as *Lycium barbarum* polysaccharides [27]. The absolute value of CM-PFP is indicative of the presence of well-dispersed polysaccharide molecules in the solution. The zeta potential results demonstrate that carboxymethylation has altered the anionic character of polysaccharides [25]. Generally, CM-PFP was more hydrophilic than PFP owing to the presence of carboxylate groups [24]. The surface of *Cordyceps militaris* polysaccharide (CPS) had an interlaced, multi-branched structure with a leaf- or chain-like shape, similar to that of PFP. Carboxymethylated modification affected the structure of CPS, causing a shift in the aggregation states [17]. A possible reason for this is the degradation of the polysaccharides during carboxymethylation, which can deconstruct the original structure. However, polysaccharides extracted from blackcurrant fruits and their carboxymethylated derivatives showed similar surface morphologies, and no new features were generated with both polysaccharides exhibiting flake-like structures with uneven surfaces [23].

### 3.3. In Vitro Proliferation of Lactobacillus Bacteria

According to the findings, the inclusion of PFP served as an efficient carbon source and facilitated the development of *Lactobacillus* strains. Furthermore, the growth rate of *Lactobacillus* in CM-PFP media was higher than that in PFP media. Introducing carboxymethyl groups affected the PFP dissolving and oxidative/reductive properties. These changes may mean that CM-PFP is utilized by *Lactobacillus* strains more efficiently than PFP. *L. acidophilus NCFM* in the logarithmic growth period from 2 h to 10 h and entered the stationary period from 10 h to 20 h in persimmon polysaccharide extracts medium compared with MRS medium [8]. After 24 h of culture, there was a significant increase in the population of *Lactobacillus*. However, after 48 h, the number of *Lactobacillus* decreased significantly. Polysaccharides from bamboo shoot (*Chimonobambusa quadrangularis*) residues showed similar prebiotic activity [28]. Jujube polysaccharides as the sole carbon source promoted the proliferation of *Lactobacillus* strain after 48 h incubation and had the best effect on bacterial population proliferation when the concentration was 2.0% [11]. Longan polysaccharides (LPs) stimulated the highest increased-fold *L. acidophilus* populations and FOS had the most beneficial effect on *L. bulgaricus* and *L. plantarum* proliferation during the tested concentrations (0.5–3.0%) [29]. After 48 h fermentation, *L. acidophilus* CCFM202 and *L. plantarum* CCFM6392 fermented water-soluble polysaccharides from Maitake fruiting body obtained the highest viable bacterial counts [10]. In conclusion, PFP and CM-PFP were nontoxic to *Lactobacillus* and promoted the growth of *Lactobacillus*. Modified persimmon polysaccharides have the potential to provide high-quality carbon sources for *Lactobacillus*, increase acid production, and maintain the health of the intestinal environment.

### 3.4. Inhibitory Effects on Escherichia coli and Staphylococcus aureus

The antibacterial activity of plant polysaccharides has been demonstrated to rise as concentrations increased in a few studies, but the degree and mechanism of antagonism were different because of the different types of polysaccharides [30]. According to a previous study by Weng et al., 5 mg/mL *Lindera aggregata (Sims) Kosterm.* leaves polysaccharides (LLPs) prevented *E. coli* and *S. aureus* growth for 6 h, the inhibitory level were 40% and 50.4% respectively [31]. Furthermore, using tapioca starch as a carbon source for polysaccharide production through static liquid fermentation had a greater inhibitory effect on *S. aureus* than on *E. coli*, which differs from the results of the present study [32]. As the main monosaccharide of CM-PFP and PFP, glucose can be used immediately by gut microbes for the rapid production of metabolites, resulting in lowering the pH of the fermenting broth. As such, the fermentation process by the intestinal flora is responsible for the lowering of the pH, such as acetic acid and propionic acid [9]. SCFAs are the major metabolites and pH-lowering factors in the fermentation process and their concentrations are key indicators of gut microbiota activity [33]. Acetic and propionic acids are the major constituents of the total amount of SCFAs produced by fermentation, and their presence has been reported to be beneficial for human health [34].

### 3.5. In Vitro Fecal Fermentation

A similar conclusion was reached in a study on fecal flora grown on rice with added FOS; at the phylum level, the microbiota studied were mostly made up of *Proteobacteria*, *Bacteroidetes*, *Firmicutes,* and *Actinobacteria* [20]. *Bacteroidetes* can hydrolyze non-digestible polysaccharides to produce SCFAs by encoding various carbohydrate-active enzymes such as glycosidases and polysaccharide lyases [35]. An increase in *Proteobacteria* leads to an imbalance in gut microbiota, inflammation, and colon cancer [36]. *Bifidobacterium*, a representative *Actinobacteria*, are essential probiotics for human intestinal health and have been reported to control serum cholesterol levels, prevent intestinal diseases, regulate the immune system, and exert anti-cancer activity [37]. *Firmicutes* have the effect of positively influencing intestinal barrier function by fermenting carbohydrates into a variety of SCFAs [8]. The increase in the *Firmicutes*/*Bacteroidetes* ratio contributes to weight gain, which is positively correlated with a risk of obesity in humans [38]. Therefore, PFP should be investigated against obesity further. According to a previous investigation by Wu, the relative abundance of *Escherichia* declined due to their inability to digest polysaccharides from *Tremella fuciformis* [21]. Meanwhile, the presence of glucose promotes the proliferation of *Bacteroides* and *Parabacteroides* [20]. *Bacteroides* may be able to metabolize diverse polysaccharides for the production of SCFAs. *Bacteroides* are beneficial for obese individuals, helping them maintain their health with metabolic and immunological disorders [39]. *Parabacteroides* exhibit a negative correlation with obesity, diabetes, and other diseases. These results indicated that CM-PFP stimulates the proliferation of beneficial bacteria and inhibits the proliferation of harmful bacteria in the gut. *Clostridium* is associated with sepsis and can lead to death and human nosocomial infections [9]. The above analysis is consistent with the results at the genus level. *Parabacteroides* have been shown to metabolize carbohydrates and secrete SCFAs [40]. *Veillonellaceae*, a core bacterial genus of the human gut, is related to metabolizing polysaccharides and SCFAs [35]. Collectively, CM-PFP exhibits potential prebiotic activities by regulating the composition and abundance of beneficial gut microbiota that can be used as functional foods.

## 4. Materials and Methods

### 4.1. Materials and Chemicals

Mopan persimmon fruit was collected from Baoding, Hebei Province, China, in October. The peel was rinsed and stored at −20 °C.

The strains *Lactobacillus acidophilus* NCFM (*L. acidophilus* NCFM), *Lactobacillus plantarum* 121(*L. plantarum* 121), *Lactobacillus helveticus* LH-10 (*L. helveticus* LH-10), *Lactobacillus bulgaricus* LB-2 (*L. bulgaricus* LB-2), *Bifidobacterium animalis* 02 (*B. animals* 02), and *Bifidobacterium breve* CICC 6185(*B. breve* CICC 6185) were purchased from the China Center of Industrial Culture Collection. Lactobacil lus strains were reactivated in De Man, Rogosa, and Sharpe broth (MRS).

### 4.2. Extraction and Purification of PFP

The PFP was prepared according to the method described by Yang and made some changes [8]. Persimmon pulp was precipitated with an 80% ethanol solution to remove monosaccharides and lipids. The pulp was then filtered using gauze, and the residue was added to distilled water at a 1:3 solid-to-liquid ratio. The mixture was subjected to hydrolysis at 45 °C for 2 h, using 3% complex enzymes (cellulase: pectinase: papain = 1:1:0.5). The supernatant was combined, heated at 95 °C for 15 min, and then filtered, concentrated under vacuum, precipitated. Finally, the crude PFP was freeze-dried.

Proteins were removed from the crude PFP using the Sevag-enzyme-linked method. To do so, the crude PFP was dissolved in distilled water, and a 2–3% papain solution was added. The mixture was subject to a hydrolytic treatment at 60 °C for 4 h. The solution was mixed with the Sevag reagent in a 5:1 volume ratio, centrifuged, and the supernatant collected. The deproteinized PFP was obtained by vacuum freeze-drying the precipitate, and its protein content was assessed by Coomassie Brilliant Blue G-250 binding [41].

An AB-8 macroporous adsorption resin was used to remove pigments from the deproteinized PFP. The wet resin was placed into a conical flask, and 20 mL of a deproteinized PFP solution (2 mg/mL) was added. The mixture was stirred at 150 rpm for 12 h at room temperature. The absorbances of the solutions before and after depigmentation were measured at 513 nm. Polysaccharide content was determined using the phenol-sulfuric acid method [42].

### 4.3. Carboxymethylation of PFP

The solvent method was used for the carboxymethylation of PFP. PFP (1.0 g) was complexed with 25 mL reagent and stirred evenly, after which 25 mL of 15% NaOH was poured in, and the mixture was stirred for 1 h. Chloroacetic acid was then dissolved and added to the solution for etherification. The solution was then neutralized with glacial acetic acid, and ethanol was added to precipitate the product for 12 h. The mixture was centrifuged and the residual material was lyophilized to yield CM-PFP.

The DS of PFP was determined according to acid-base titration [22]. CM-PFP (10 mg) was added to 10 mL of standard HCL solution (0.1 mol/L) and then titrated with a standard NaOH solution (0.1 mol/L). The volume of NaOH consumed was measured at pH 2.1 and pH 4.3.

Single-factor experiments were used to explore five different carboxymethylation conditions and how they affected the DS. The solvent (water, ethanol, isopropanol), the temperature of alkalization (40 °C, 45 °C, 50 °C, 55 °C, and 60 °C), chloroacetic acid dosage (0.50, 0.75, 1.00, 1.25, and 1.50 g), temperature of etherification (60 °C, 65 °C, 70 °C, 75 °C, and 80 °C), and time of etherification (2.5 h, 3 h, 3.5 h, 4 h, and 4.5 h) were all investigated. Based on the single-factor experiments, the temperature of alkalization, the dosage of chloroacetic acid, the temperature of etherification, and the time of etherification were selected as factors, and the DS was used as the evaluation index. An orthogonal L_9_(3^4^) experiment was designed to optimize the experimental conditions for carboxymethyl modification (Table S1a).

### 4.4. Physicochemical Properties and Structural Characterization of PFP and CM-PFP

#### 4.4.1. The FTIR Spectra of PFP and CM-PFP Analysis

In a dry environment, PFP and CM-PFP were added to a mortar containing dried potassium bromide powder and ground thoroughly before being pressed into tablets. FTIR spectra of PFP and CM-PFP samples were obtained using an FTIR spectrometer (Nicolet iS20, Thermo Fisher Scientific, Waltham, MA, USA), scanning over the wavenumber range from 400 to 4000 cm^−1^.

#### 4.4.2. Measurement of Mw of PFP and CM-PFP

The Mw of PFP and CM-PFP were analyzed by using high-performance gel permeation chromatography (HPGPC). HPGPC analysis was conducted with a GPC (Waters 1515 GPC, Waters Corporation, Milford, MA, USA) coupled with a refractive index detector (Shodex SB-806M, Shodex, Tokyo, Japan) equipped with a chromatographic column (SB-802.5HQ). Sodium chloride and disodium hydrogen phosphate were used as mobile phases. The flow rate was 0.8 mL/min. The column temperature was 25 °C. Before analysis, the PFP and CM-PFP solutions (2 mg/mL) were filtered using a 0.2 μm filter, and 100 μL of the sample was injected.

#### 4.4.3. Monosaccharide Composition of PFP and CM-PFP

Briefly, PFP or CM-PFP was dissolved in 2 mol/L trifluoroacetic acid and incubated at 121 °C for 2 h. The samples were then dried under nitrogen, cleaned by adding 99.99% methanol, and dried once more. The samples were then re-dissolved in sterile water for testing. Thirteen monosaccharide standard solutions fucose (Fuc), rhamnose (Rha), arabinose (Ara), galactose (Gal), glucose (Glc), xylose (Xyl), mannose (Man), fructose (Fuc), ribose (Rib), galacturonic acid (GalA), glucuronic acid (GlcA), mannuronic acid (ManA), guluronic acid (GulA) were processed as described above. The series of standard products required by the machine was prepared by diluting different concentration gradients. The monosaccharide compositions of PFP and CM-PFP were determined by high-performance ion exchange chromatography (HPIEC) (Dionex ICS-5000, Thermo Fisher Scientific, Waltham, MA, USA) [26]. Finally, monosaccharide components of supernatant were analyzed by HPIEC equipped with a liquid chromatography column (Dionex™ CarboPac™ PA20 (150 × 3.0 mm, 10 μm), Thermo Fisher Scientific, Waltham, MA, USA). The injection volume was set at 5 μL. Mobile Phase A was H_2_O, Mobile Phase B was 0.1 mol/L NaOH, and Mobile Phase C was a combination of 0.1 mol/L NaOH and 0.2 mol/L NaAc. The flow rate was 0.5 mL/min. The column temperature was 30 °C.

#### 4.4.4. X-ray Diffraction (XRD) Analysis

The crystalline properties of both PFP and CM-PFP were measured using an X-ray diffractometer (Ultima IV, B Rigaku Corporation, Tokyo, Japan). The diffraction conditions were a copper target, a current of 27 mA, and a voltage of 44 kV. The prepared samples were placed on a tray with a scanning range of 5–45°. The scanning speed was set at 5 °/min. MDI Jade software (version 6.0) was used to analyze the XRD patterns of PFP and CM-PFP.

#### 4.4.5. Zeta Potential Determination

Solutions of PFP and CM-PFP, at a concentration of 0.003% (*w*/*v*), were prepared, and their potential was determined (Zetasizer Nano ZS90, Malvern Instrument, Malvern, UK) at 25 °C.

#### 4.4.6. Thermal Analysis

TG-DSC analysis of PFP and CM-PFP (5 mg) was performed using a Mettler TGA/DSC (TA Q600, Thermo Fisher Scientific, Waltham, MA, USA; DSC 8000, Perkin Elmer Instruments Co., Ltd., Waltham, MA, USA). PFP and CM-PFP samples (5 mg) were heated from 30 to 600 °C (10 °C/min), and the nitrogen flow rate was 40 mL/min.

#### 4.4.7. SEM Analysis

PFP and CM-PFP samples were prepared using the ion sputtering coating method and then placed in the sample chamber of the SEM for scanning and analysis. The acceleration voltage was adjusted to 10 kV, and the magnification was ×5000.

### 4.5. In Vitro Proliferation of Lactobacillus Bacteria

The proliferation of *Lactobacillus* strains was tested in a different type of carbon source of MRS medium, respectively, containing glucose, PFP, CM-PFP, or fructooligosaccharides (FOS). The Carbohydrate-free MRS was supplemented with 0.05% (*w*/*v*) L-Cys as basal medium (control). Glucose (2.0%, *w*/*v*) was added to the basal medium as a positive control (Glc group), and PFP and CM-PFP (0.1%, *w*/*v*) were added to the medium, respectively. The basal medium supplemented with FOS (2.0%, *w*/*v*) was used as the prebiotic reference. *Lactobacillus* bacteria powders were inoculated (5%) in MRS at 37 °C for activation. *B. animals* 02 and *B. breve* CICC 6185 were cultured in an anaerobic tank and sub-cultured three times every 12 h. The activated *Lactobacillus* were cultured at 37 °C for 48 h. *Lactobacillus* were extracted at 0 h, 24 h, and 48 h after fermentation, and the absorbance at OD_600_ and pH changes were measured. Growth curves were plotted using culture time as the *x*-axis and OD_600_ as the *y*-axis.

### 4.6. Inhibitory Effects of PFP and CM-PFP on E. coli and S. aureus

*E. coli* and *S. aureus* were activated in Lysogeny broth (LB) medium cultured at 37 °C. The bacteria were sub-cultured three times every 12 h to restore their activity. PFP and CM-PFP were added to the LB medium, and the final concentrations of polysaccharides used were 2, 4, 6, and 8 mg/mL. LB medium was chosen as the control and FOS as the positive control. *E. coli* and *S. aureus* were cultured to the logarithmic growth phase, inoculated into 5 mL of different medium, and incubated at 37 °C. The absorbance of the bacterial solution at a wavelength of 600 nm was measured at 0 h, 1 h, 2 h, 4 h, 6 h, 8 h, and 10 h, respectively.

### 4.7. In Vitro Fecal Fermentation

#### 4.7.1. Analysis of SCFA Production Ability

Fresh fecal samples were obtained from three healthy donors who had never been diagnosed with bowel disease and who followed normal diets without any history of antibiotic use, other medication, or dietary supplements for at least three months [34]. All volunteers were informed about the experimental content and the publication of the article groups. The fecal sample was diluted with a sterile PBS buffer containing 0.5 mg/L VB_1_ and 0.5 mg/L VB_2_ to obtain a 10% (m/m) solution and homogenized. To obtain the human fecal inoculum, the suspension was collected by centrifugation (1000× *g* rpm) for 5 min.

The fermentation medium was prepared and sterilized at 121 °C for 30 min. The carbohydrate-free medium served as the control, inulin-treated media as the positive control, PFP- and CM-PFP-treated media as the experimental groups. In vitro, fermentation was conducted at 37 °C for 48 h in an anaerobic environment. Specifically, a sterile test tube was placed with 0.3 mL of fecal suspension and 2.7 mL of growth medium. Samples were removed and placed in an ice-water bath for 20 min at 0 h, 6 h, 12 h, 24 h, 36 h, and 48 h to terminate fermentation. The supernatant was collected by centrifugation (8000× *g* rpm for 15 min), and the pH of the supernatant was measured. The sediment was collected in the −80 °C. Using the method described by Yang, short-chain fatty acids (SCFAs) in feces were detected using HPLC [8].

#### 4.7.2. 16S rRNA Analysis

The collected sediment above was used for 16S rRNA sequencing of the microbiota. Experimental procedures: sample preparation, DNA extraction and PCR amplification, and Illumina Miseq sequencing were all carried out as previously described [8].

### 4.8. Statistical Analysis

All experiments were conducted in triplicate. The relative standard deviation (RSD) of the detection results was calculated, and the experimental data were expressed as the mean ± standard deviation. Statistical analyses were performed using software (Origin 2021, OriginLab Corporation, Northampton, MA, USA). Statistical significance was determined using ANOVA. Differences were considered statistically significant when *p* < 0.05.

## 5. Conclusions

Mopan persimmon polysaccharide was modified with carboxymethyl groups. The structures of Mopan PFP and CM-PFP were comprehensively analyzed, and the effect of PFP on human intestinal flora was explored. Through XRD and HPGPC analysis, it was determined that carboxymethylation increased the Mw and Mn but did not change the backbone and crystal structures. Fourier transform infrared analyses indicated that PFP is predominantly composed of pyran-type sugars. Furthermore, the HPIEC test revealed that the monosaccharide compositions of PFP and CM-PFP remained the same, with glucose likely forming the backbone of the main chain. The zeta potential and TG-DSC results proved that carboxymethylation altered the dispersion and thermal stability of the polysaccharides. CM-PFP was dispersed and thermally degraded more easily than PFP. The proliferative effect of CM-PFP on *Lactobacillus* was greater than that of PFP and FOS. In addition, the relative abundances of harmful intestinal bacteria, such as *Escherichia*, *Enterobacter*, *Citrobacter*, and *Clostridium*, were significantly reduced in the PFP and CM-PFP.

## Figures and Tables

**Table 1:** Chemical composition of crude PFP, deproteinization PFP, PFP, CM-PFP.

Sample	Total Sugar Content (%)	Content of Protein (%)	L*	a*	b*
Crude PFP	42.8 ± 0.21	11.8 ± 0.12	7.24	77.30	11.90
Deproteinized PFP	64.3 ± 0.33	3.1 ± 0.04	18.68	50.19	24.28
PFP	77.24 ± 0.07	2.9 ± 0.41	30.80	20.17	23.19
CM-PFP	76.58 ± 0.22	2.8 ± 0.03	32.63	18.90	21.86

Note: L* indicates the brightness of the color, with positive numbers indicating white and negative numbers indicating black. a* indicates red-green values, with positive numbers indicating more red and negative numbers indicating much green. b* indicates yellow and blue values; positive numbers indicate yellow and negative numbers indicate blue.

**Table 2:** The Mw of PFP and CM-PFP Monosaccharide components of PFP and CM-PFP.

Sample	0–5000 Da	5000–10,000 Da	10,000–20,000 Da	Mw	Mn	PD (Mw/Mn)
PFP	51.668%	18.868%	18.132%	13,327 Da	2164 Da	6.126
CM-PFP	8.767%	64.506%	35.064%	16,566 Da	9502 Da	1.743

**Table 3:** The monosaccharide components of PFP and CM-PFP.

Monosaccharides	PFP	CM-PFP
Ara	0.23%	0.10%
Gal	0.38%	0.16%
Glc	95.62%	97.61%
Man	0.99%	0.62%
Rib	0.00%	1.08%
GalA	1.54%	0.18%
GulA	0.66%	0.25%
GlcA	0.58%	0.00%

## Data Availability

The data presented in this study are available on request from the corresponding author.

## Supplementary Materials

The following supporting information can be downloaded at: https://www.mdpi.com/article/10.3390/ijms242115730/s1.
