# Supplementary material for: Effects of Persimmon (Diospyros kaki L. cv. Mopan) Polysaccharide and Their Carboxymethylated Derivatives on Lactobacillus Strains Proliferation and Gut Microbiota: A Comparative Study
Source: Int J Mol Sci. 2023 Oct 29;24(21):15730. doi: 10.3390/ijms242115730 (PMC10648239; doi:10.3390/ijms242115730)
Supplement: Supplementary file 1 [file ijms-24-15730-s001.zip › ijms-2663480-supplementary.pdf]

Table S1 (a) Orthogonal experimental results; (b)Analysis of variance of orthogonal test results

a

Level	Factor			
	A<br>Alkalization temperature/°C	B<br>Dosage of chloroacetic acid /g	C<br>Etherification temperature/°C	D<br>Etherification time/h
1	40	0.75	55	2.5
2	45	1	60	3
3	50	1.25	65	3.5

Group	A Alkalization temperature (°C)	B Chloroacetic acid dosage (g)	C Etheration temperature (°C)	D Etherification time (h)	DS
1	1	1	1	1	0.495
2	1	2	2	2	0.453
3	1	3	3	3	0.496
4	2	1	2	3	0.467
5	2	2	3	1	0.281
6	2	3	1	2	0.488
7	3	1	3	2	0.278
8	3	2	1	3	0.503
9	3	3	2	1	0.374
k1	0.481	0.423	0.433	0.426	
k2	0.412	0.424	0.418	0.411	
k3	0.385	0.412	0.402	0.419	
R	0.096	0.011	0.031	0.016	
Optimal level	$A_1B_2C_1D_1$				
Degree of influence	A>C>D>B				

b

Factor	Sums of squared deviations	degrees of freedom	Mean square	F	Sig.	Statistical significance
Model modification	0.197a	8.000	0.025	38.341	0.000	
Intercede	4.642	1.000	4.642	7216.369	0.000	
Alkalization temperature	0.040	2.000	0.020	31.350	0.000	**
Chloroacetic acid dosage	0.009	2.000	0.005	7.290	0.005	**
Etheration temperature	0.087	2.000	0.043	67.578	0.000	**
Etherification time	0.052	2.000	0.026	40.686	0.000	**
Errors	0.011	17.000	0.001			
Sum	4.868	26.000				

Table S2 (a) The proliferation rate of selected *Lactobacillus* in different carbon source media varies with fermentation time (Different lowercase letters indicate significant differences (P＜0.05)); (b) Culture medium pH for different *Lactobacillus* and carbon sources after fermentation for 48 h (Different lowercase letters indicate significant differences (P＜0.05); (c) Growth inhibition rate of *E. coli* and *S. aureus* by different carbon sources.

a

Strains	Group	PI (%) 0-24h	PI (%) 24-48h	PI (%) 0-48h
L. acidophilus NCFM	Control	0.638 ± 0.028^{d}	0.170 ± 0.031^{a}	0.917 ± 0.032^{d}
	Glc	5.850 ± 0.013^{c}	0.033 ± 0.003^{b}	6.077 ± 0.016^{c}
	FOS	6.478 ± 0.171^{a}	-0.040 ± 0.002^{c}	6.181 ± 0.177^{bc}
	PFP	6.263 ± 0.047^{b}	0.012 ± 0.002^{b}	6.350 ± 0.036^{b}
	CM-PFP	6.552 ± 0.043^{a}	0.000 ± 0.001^{b}	6.556 ± 0.040^{a}
L. plantarum 121	Control	0.289 ± 0.002^{c}	-0.017 ± 0.007^{b}	0.267 ± 0.009^{c}
	Glc	5.107 ± 0.087^{b}	-0.008 ± 0.001^{b}	5.060 ± 0.090^{b}
	FOS	6.090 ± 0.281^{a}	0.015 ± 0.003^{a}	6.199 ± 0.288^{a}
	PFP	6.074 ± 0.066^{a}	-0.014 ± 0.003^{b}	5.975 ± 0.056^{a}
	CM-PFP	6.295 ± 0.095^{a}	-0.014 ± 0.002^{b}	6.191 ± 0.096^{a}
L. helveticus LH-10	Control	0.162 ± 0.007^{c}	-0.035 ± 0.004^{d}	0.121 ± 0.008^{b}
	Glc	4.925 ± 0.003^{b}	0.008 ± 0.001^{c}	4.970 ± 0.003^{ab}
	FOS	6.335 ± 0.337^{a}	0.016 ± 0.047^{a}	6.444 ± 0.016^{ab}
	PFP	5.963 ± 0.052^{a}	0.027 ± 0.001^{b}	6.155 ± 0.061^{a}
	CM-PFP	6.143 ± 0.041^{a}	-0.003 ± 0.002^{b}	6.121 ± 0.051^{ab}
L. bulgaricus LB-2	Control	0.543 ± 0.004^{e}	0.070 ± 0.005^{a}	0.651 ± 0.013^{d}
	Glc	6.668 ± 0.026^{c}	-0.031 ± 0.001^{d}	6.427 ± 0.024^{c}
	FOS	6.391 ± 0.007^{d}	0.029 ± 0.003^{b}	6.604 ± 0.019^{b}
	PFP	6.803 ± 0.014^{b}	-0.022 ± 0.001^{c}	6.632 ± 0.008^{b}
	CM-PFP	6.961 ± 0.023^{a}	-0.027 ± 0.001^{cd}	6.749 ± 0.016^{a}
B. breve CICC 6185	Control	0.480 ± 0.024d	-0.072 ± 0.019c	0.374 ± 0.007e
	Glc	6.284 ± 0.240c	-0.041 ± 0.002b	5.982 ± 0.232d
	FOS	6.388 ± 0.038c	0.005 ± 0.002a	6.422 ± 0.038c
	PFP	9.524 ± 0.134b	-0.026 ± 0.002b	9.254 ± 0.146b
	CM-PFP	9.907 ± 0.245a	-0.025 ± 0.001b	9.636 ± 0.241a
B. animals 02	Control	0.325 ± 0.024d	0.210 ± 0.005a	0.604 ± 0.024c
	Glc	6.066 ± 0.117b	-0.054 ± 0.007d	5.686 ± 0.119b
	FOS	5.565 ± 0.167c	-0.008 ± 0.001b	5.513 ± 0.160b
	PFP	6.303 ± 0.022a	-0.028 ± 0.001c	6.095 ± 0.025a
	CM-PFP	6.350 ± 0.008a	-0.032 ± 0.001c	6.110 ± 0.008a

b

Medium	*L. acidophilus* NCFM	*L. plantarum* 121	*L. helveticus* LH-10	*L.bulgaricus* LB-2	*B. Animals* 02	*B.breve* CICC 6185
Control	5.813±0.015^{a}	6.010±0.010^{a}	5.830±0.010^{a}	5.844±0.045^{a}	5.710±0.015^{a}	5.827±0.025^{a}
Glu	3.943±0.038^{b}	3.827±0.025^{d}	4.388±0.169^{b}	4.048±0.040^{c}	3.727±0.010^{c}	3.730±0.010^{c}
FOS	3.730±0.020^{c}	4.090±0.026^{b}	4.162±0.060^{c}	4.136±0.017^{b}	3.823±0.032^{b}	4.033±0.023^{b}
PFP	3.920±0.020^{b}	3.910±0.010^{c}	4.160±0.026^{c}	3.918±0.017^{d}	3.710±0.025^{c}	3.723±0.006^{c}
CM-PFP	3.913±0.006^{b}	3.623±0.015^{e}	3.928±0.021^{d}	4.035±0.036^{c}	3.723±0.015^{c}	3.730±0.026^{c}

c

Carbon source	*E. coli*	*S. aureus*
FOS	5.721±0.196	-6.756±0.059
PFP	28.83±0.193	12.744±0.202
CM-PFP	14.622±0.195	18.536±0.038
